# Epidemiology of respiratory viral infections in children enrolled in a study of influenza vaccine effectiveness

**DOI:** 10.1111/irv.12229

**Published:** 2014-01-31

**Authors:** Alexa Dierig, Leon G Heron, Stephen B Lambert, Jiehui Kevin Yin, Julie Leask, Maria Yui Kwan Chow, Theo P Sloots, Michael D Nissen, Iman Ridda, Robert Booy

**Affiliations:** a National Centre for Immunisation Research and Surveillance, The Children's Hospital at WestmeadWestmead, NSW, Australia; b University Children's Hospital both BaselBasel, Switzerland; c Sydney Medical School, The University of SydneySydney, NSW, Australia; d Marie Bashir Institutesydney, NSW, Australia; e Queensland Paediatric Infectious Disease Laboratory, Queensland Children's Medical Research Institute, Queensland Children's Health ServiceBrisbane, Qld, Australia; f Clinical and Statewide Services, Pathology Queensland CentralHerston, Qld, Australia

**Keywords:** Children, influenza, respiratory viral infections

## Abstract

**Background:**

Influenza-like illness (ILI) confers a high annual morbidity in young children. We report the epidemiology of ILIs in children who participated in an influenza vaccine effectiveness study during the 2010 Southern Hemisphere influenza season in Sydney, Australia.

**Methods:**

Children aged 0·5–3 years were prospectively recruited from child care centres (CCCs). We classified them as fully vaccinated, partially vaccinated and unvaccinated according to their receipt of unadjuvanted vaccines containing influenza A (H1N1)pdm09. For 13 weeks commencing 30 July 2010, parents reported when their children developed an ILI (fever ≥37·8°C/feverishness plus ≥1 respiratory symptom) and collected nose and/or throat swabs for multiplex respiratory virus polymerase chain reaction (PCR) testing. Health impacts were assessed by telephone interview at enrolment and two weeks after each ILI.

**Results:**

There were 124 ILIs reported in 105 of 381 enrolled children. Swabs were taken in 117 ILIs: 175 viruses were identified from 103 swabs. Adeno- and rhinoviruses were most frequently identified; 44% of swabs yielded multiple viruses. No virus was associated with more severe symptoms, although rhinovirus-related ILIs lasted longer. Nose swabs had a higher virus detection rate than throat swabs. Influenza-vaccinated children were 1·6 times (*P* = 0·001) more likely than unvaccinated children to have a non-influenza ILI.

**Conclusion:**

Adeno- and rhinoviruses were the most common viruses causing ILI. Swabs taken by parents are an effective method for sample collection. Influenza-like illness was more common in children vaccinated against influenza in this observational study, but prior health-seeking behaviour may have contributed to this difference.

## Introduction

Acute respiratory infection (ARI) is among the major causes of death in young children worldwide.^1^ In Australia, ARI is the main cause for short-term illness in children aged 0–14 years.^2^ The number of newly identified viruses in respiratory tract specimens, including the recently discovered polyomaviruses WUV and KIV,^3^,^4^ is increasing.

Influenza causes a substantial health burden with direct and indirect costs, including hospitalisations and loss of productivity.^5^–^8^ Inactivated and live-attenuated influenza vaccines offer both direct and herd benefits to vaccinated children, their contacts and the broader community.^9^–^11^ Several studies have shown that children attending child care centres (CCCs) are at greater risk of ARI including influenza.^12^–^15^ In 2008, formal child care was undertaken by 9% of Australian children aged <1 year, 35% aged 1 year and 47% aged 2–3 years.^16^

In order to determine the health, social and economic effects of influenza vaccination in young children, we planned a randomised controlled trial (RCT) of an unadjuvanted trivalent influenza vaccine in children aged 6–35 months who attended a CCC in metropolitan Sydney during 2010. However, because of the 2009 pandemic, the Australian government recommended and funded universal use of inactivated pandemic influenza A(H1N1)pdm09 vaccination for those aged >6 months in 2010. Hence, a RCT design became unethical. The study was restructured to a prospective cohort design addressing the epidemiology of ILIs among young children.

## Methods

### Study cohorts

Children were recruited through 90 CCCs and one general practitioner with a special interest in paediatrics. Informed consent was obtained from a parent or legal guardian. To meet inclusion criteria, children needed to be aged ≥6–35 months on 1 March 2010. Exclusions were for known allergy to any component of the influenza vaccine, a history of Guillain–Barre syndrome, a bleeding disorder, an unstable chronic illness or enrolment in another trial. Parents reported influenza vaccinations their children had received and, where possible, the influenza vaccination status was validated from vaccination records. The children were divided into three cohorts: fully vaccinated (usually two doses in 2010), partially vaccinated (usually one dose in 2010) and unvaccinated according to their receipt of vaccines that contained influenza A (H1N1)pdm09 – full definitions are in the footnotes to Table 1.

**Table 1:** Demographics of enrolled children and their households and ILI outcomes by subject's influenza vaccination status

A(H1N1)pdm09 vaccination status*	Fully vaccinated** *n* = 91	Partially vaccinated*** *n* = 52	Not vaccinated† *n* = 238	*P* value
Enrolled children				
Gender male, *n* (%)	51 (56·0)	30 (57·7)	127 (53·4)	0·81
Mean age at 30 July 2010 (years)	2·30	2·24	2·29	0·54
Breastfed	85 (93)	48 (92)	213 (89)	0·25
Past medical history (PMH) hospitalisation, *n* (%)	31 (34)	17 (33)	52 (22)	0·01
PMH pneumonia, *n* (%)	5 (5)	4 (8)	12 (5)	0·77
PMH otitis media, *n* (%)	39 (43)	14 (27)	81 (34)	0·22
PMH grommet surgery, *n* (%)	6 (7)	2 (4)	3 (1)	0·01
PMH hearing test, (%)	11 (12)	4 (8)	12 (5)	0·02
Mean no. of siblings in household	0·92	0·94	0·89	0·42
Ethnicity, *n* (%)				
White	73 (82·2)	45 (86·5)	195 (81·9)	0·85
Asian	14 (15·2)	5 (9·6)	18 (7·6)	0·04
Mid-Eastern	0	0	8 (3·4)	0·04
Maori/Pacific Islander	0	0	3 (1·3)	0·21
Others	4 (4·4)	2 (3·8)	12 (5·0)	0·76
Missing	0	0	2 (0·8)	0·30
Childcare attendance, *n* (%)	79 (86·8)	46 (88·5)	215 (90·3)	0·64
Mean days at child care/week	2·72	2·95	2·84	0·28
Sleeping alone, *n* (%)	58 (63·7)	39 (75·0)	175 (73·5)	0·50
Children's households				
Accommodation, *n* (%)				
House††	76 (83·5)	46 (88·5)	217 (91·2)	0·17
Others	15 (16·5)	6 (11·5)	21 (8·8)	
Smoker(s) in household, *n* (%)	14 (15·4)	8 (15·9)	33 (13·8)	0·59
Parents' employment, *n* (%)				
Both working	64 (70·3)	45 (86·5)	169 (71·0)	
One working	27 (29·7)	7 (13·5)	65 (27·3)	
Neither working	0	0	4 (1·7)	
Parents' education††† (two-parent households n (%)‡				
Both university	43 (48)	34 (65)	108 (47)	0·44
Both other	20 (22)	6 (12)	46 (20)	0·78
One university, one other	24 (27)	11 (21)	69 (30)	0·51
Either unknown	2 (2)	1 (2)	6 (3)	0·83
Both unknown	0 (0)	0 (0)	1 (0)	0·47
Parents' education††† (single-parent households *n* (%)				
University	2 (100)	0	2 (25)	0·13
Other	0 (0)	0	6 (75)	
Household income >$2000 per week, *n* (%)	59/85 (69)	44/52 (85)	161/214 (75)	0·47
Reported ILI events, *n*	37	23	64	0·01‡‡
ILIs with measured fever ≥37·8°C	27	16	41	0·35
Influenza-positive ILI, *n*	1	1	3	
Non-influenza ILI (includes ‘no viruses detected’) (*n*)	34	19	59	
Rate (per person-year)	1·59	1·54	0·99	0·001‡‡
Virus other than influenza virus detected (*n*)	28	18	52	
Rate (per person-year)	1·23	1·38	0·87	0·02‡‡

There were eight subjects vaccinated late, and for the incidence rate calculations, their person-time contributions to the partially and fully vaccinated cohorts have been determined using the date of receipt of the second dose of vaccine as the time-point at which they changed between the cohorts.

(ILL), influenza-like illness

* Vaccination status classified according to the group in which the subjects spent the most time during follow-up.

** Fully vaccinated against A(H1N1)pdm09 (vaccinated with either 2 doses of pandemic A(H1N1)pdm09 vaccine (Panvax, CSL, the only pandemic vaccine used in Australia) in 2010, or 1 dose of Panvax and 1 dose of trivalent influenza vaccine (TIV) in 2010, or 1 dose of Panvax in 2010 and 2 doses of TIV in 2009, or 2 doses of TIV 2010, or 1 dose of TIV 2010 if the child had received 2 doses of a TIV in a previous year).

*** Partially vaccinated against A(H1N1)pdm09 (vaccinated with either 1 dose of Panvax in 2009 or 2010, 2 doses of Panvax in 2009, 1 dose of TIV 2010 if the child had never received 2 doses in one previous year).

† Not vaccinated against A(H1N1)pdm09 (no doses of TIV 2010 and no doses of Panvax in 2009 or 2010). [Based upon reference Australian Technical Advisory Group on Immunisation. Use of pandemic and seasonal influenza vaccines in children <10 years of age. January 2010.]

Southern Hemisphere TIV 2009 contained influenza A/Brisbane/59/2007 (H1N1)-like virus; influenza A/Brisbane/10/2007 (H3N2)-like virus; influenza B/Florida/4/2006-like virus.

Southern Hemisphere TIV 2010 contained influenza A/California/7/2009 (H1N1)-like virus; influenza A/Perth/16/2009 (H3N2)-like virus; influenza B/Brisbane/60/2008-like virus.

†† Detached house.

††† University versus lower level of education.

‡ More than one child per household enrolled.

‡‡ Poisson regression for rates.

### ILI case reporting

We planned to commence ILI case reporting as soon as an upswing in influenza cases in Sydney was recognised through laboratory surveillance. During the ILI follow-up period, parents were asked to report to us whenever a subject child developed an ILI, defined as fever ≥37·8°C or feeling feverishness according to the carer's assessment, plus at least one of the following symptoms: cough, rhinorrhoea/nasal congestion, sore throat. During the ILI-reporting period, each family received a weekly e-mail, text message or telephone call to remind them to contact the study team immediately if a child developed an ILI. Before the ILI-reporting period, e-mail addresses and mobile telephone numbers of all parents/guardians were confirmed with parents/guardians to ensure that they received messages. In addition, during the 1 week of the ILI-reporting period, all parents/guardians were contacted/recontacted until they indicated that they had received the message that the ILI-reporting period had commenced. Parents/guardians were provided with plastic shaft rayon-budded swabs and plastic transport tubes with a foam pad reservoir soaked in viral transport medium (Virocult MW950; Medical Wire & Equipment, Wiltshire, UK). They were given verbal and written instructions on how to collect a nose swab and a throat swab from the subject whenever an ILI occurred. We asked for a nose swab to be done first and did not insist on a throat swab if the parents felt uncomfortable to collect one. Parents mailed swabs to the Queensland Paediatric Infectious Diseases Laboratory (QPID), where they were stored at −80°C until tested.

### Laboratory methods

Swabs were tested for 19 respiratory viruses by qualitative real-time PCR,^17^–^19^ including influenza viruses A and B (Flu A, Flu B), adenovirus (AV), human rhinovirus (HRV), polyomaviruses (JCV, BKV, WUV, KIV), parainfluenza viruses 1, 2, 3 (PIV1, PIV2, PIV3), coronaviruses (HCoV-OC43, HCoV-NL63, HCoV-229E, HCoV-HKU1), human metapneumovirus (HMPV), bocavirus (BV) and human respiratory syncytial viruses A and B (hRSV A, hRSV B). All RNA virus assays used Qiagen One-Step RT-PCR, Qiagen (Melbourne, Victoria, Australia), and all DNA virus assays used Qiagen Quantitect Probe PCR Mix, Qiagen, Australia. In order to assess extraction quality, specimens were spiked with equine herpes virus-1 (EHV-1) and tested for EHV-1 using a duplex real-time PCR assay. Any samples that failed the EHV-1 quality control were re-extracted. In order to monitor quality of specimen collection, specimens were tested for human endogenous retrovirus 3 (ERV3) using a duplex real-time PCR assay. In addition, the positive controls included in each PCR run were monitored for any shift in cycle threshold values to detect problems within individual runs.

### ILI outcome assessment

In order to determine health impacts of the ILI event upon the child and the household, study staff interviewed the child's parent/guardian by telephone 2 weeks after the onset of each ILI in subject children, and if, at that time, the subject child still had ILI symptoms other than a dry cough, another telephone interview was arranged and conducted 2 weeks later. Data collected included the nature and duration of symptoms, severity of illness, intrahousehold spread, visits to healthcare providers and medication usage. Severe ILIs were defined as having at least one of the following features: fever ≥5 days, any symptom other than dry cough persisting more than 14 days, otitis media, suspected bacterial respiratory infection or admission to hospital.

### ILIs in household members

During ILI outcome assessment interviews, parents were asked to report ILIs in household members in the week before and the week after the onset of an ILI in the subject children. ILI attack rates in household members were calculated for the week following ILI onset in subject children.

### Statistical analysis

Statistical tests used were one-way anova for continuous variables, chi-square tests for categorical data (SPSS 19, Chicago, IL, USA) and t-tests for normally distributed data to compare means.

Poisson regression (STATA/SE 12·0, StataCorp LP, TX, USA) was used to compare incidence rates. We used person-year in the calculation. For children who became (fully) vaccinated in the few days after the formal start date of follow-up (30 July 2010), we deducted the number of days until the children became (fully) vaccinated from the surveillance time.

Meta-Analyst 3·13 (Tufts Medical Centre, Boston, MA, USA) was used to calculate the probability of a virus being the sole agent identified from nose/throat swabs during an ILI episode (binary analysis with model type Random (D/L) and random method Der-Simonian Laird).

### Ethical approval

The study was approved by the Human Research Ethics Committee at The Children's Hospital at Westmead and was registered with the Australian New Zealand Clinical Trials Registry (ANZCTR, ACTRN12610000319077).

## Results

### The cohorts

Between March and August 2010, we enrolled 399 children, of which 95·6% completed follow-up (exclusions: 9 were of incorrect age and nine others withdrew without contributing to the ILI-reporting period); therefore, 381 children (208 males) from 358 households participated. The ILI-reporting period was 30 July to 31 October 2010. There were just four subjects who were enrolled slightly late during the first week of the ILI-reporting period – their person-year contribution to the ILI follow-up period was adjusted to be from the time of joining the cohorts (one was fully vaccinated, one partially vaccinated and two unvaccinated). The majority (89%) of enrolled children attended CCCs. On commencement of ILI surveillance, the mean age of enrolled children was 2·3 years (0·9–3·4 years). At that time, 83 (22%) children fulfilled the criteria for ‘fully vaccinated’ against influenza A/California/7/2009 (H1N1) (A(H1N1)pdm09); 60 (16%) were ‘partially vaccinated’, and 238 (62%) were unvaccinated (see Table 1). The great majority given influenza vaccine (94·4%) completed vaccination by the beginning of the formal ILI follow-up period; eight subjects received their second dose of influenza vaccine after follow-up began (between 1 August and 3 September). These subjects were primarily assigned to the fully vaccinated cohort, and for incidence rate calculations, their person-time contributions to the partially and fully vaccinated cohorts were determined using 1 week after the date of receipt of the second dose of vaccine as the time-point at which they changed status.

All vaccines given were licensed unadjuvanted inactivated split virion vaccines. There were no demographic differences between the vaccinated children, partially vaccinated children and unvaccinated children (Table 1).

### ILI episodes

During the 13 weeks 30 July to 31 October 2010, parents/guardians reported a total of 124 ILI episodes in 105 children (13 had two ILIs, three had three). Symptomatic ILIs were reported significantly more commonly in recipients of influenza vaccination (Table 1). Non-influenza ILIs were more common among fully vaccinated subjects (33 non-influenza ILIs, 1·59/person-year of observation) and partially vaccinated subjects (20 non-influenza ILIs, 1·54/person-year) than among unvaccinated subjects (59 non-influenza ILIs, 0·99/person-year, *P* = 0·001, rate ratio 1·6, vaccinated versus unvaccinated, Table 1). Excluding ILIs from which no virus was identified made no significant difference to this finding. No particular respiratory virus, with the exception of AV, was found less frequently in ILI episodes among unvaccinated subjects compared to fully or partially vaccinated subjects (*P* = 0·04, data not shown). The vaccination status of subjects was not correlated with the mean number of doctor (GP, emergency department or specialist) visits made in response to non-influenza ILIs (*P* = 0·45, data not shown). Nor were there significant differences in the mean duration of ILIs (*P* = 0·95) or use of antibiotics (*P* = 0·92) for non-influenza ILIs between fully or partially vaccinated subjects and unvaccinated subjects (data not shown). However, before enrolment in the study, there was evidence for an increased use of healthcare services in both the partially and fully vaccinated groups with significantly higher rates of prior hospitalisation, hearing tests and grommet insertion, whereas the incidence of past otitis media was not significantly different between the groups (Table 1). Increased use of these healthcare services did not, however, prove significant when added to a multivariate model to predict non-influenza ILI in study subjects (data not shown).

The apparent greater risk of non-influenza ILI in influenza-vaccinated subjects did not vary significantly over time, compared with non-vaccinated participants. For example, the rates of non-influenza ILI between the two groups (vaccinated versus non-vaccinated) were 1·88/person-year and 1·09/person-year, respectively (rate ratio = 1·72, *P* < 0·001) in the first half of follow-up period, while the rates in the second half were 1·40/person-year and 0·97/person-year, respectively (rate ratio = 1·45, *P* = 0·02).

### Follow-up of ILIs

Telephone follow-up 2 weeks after ILI onset was conducted for all 124 ILIs. Symptoms other than dry cough persisted in 29 ILIs at that time. At the 4-week telephone interview, symptoms other than dry cough still persisted in eight ILIs. Data on ILI duration were not available for five ILIs. The most commonly reported symptoms (data available for 121 of the 124 episodes) were rhinorrhoea 92% (111), cough 63% (76), decreased activity 27% (33), gastrointestinal symptoms 22% (abdominal pain, diarrhoea, vomiting; 27) and sore throat 20% (24). Fever was documented in 84 ILIs (68%). The mean temperature was 38·7°C. The frequency of documented fevers was similar in each of the three cohorts – see Table 1. Managing the ILIs required 134 GP visits (for 70 ILIs, 35 of which required more than one GP visit), 106 pharmacy visits (64 ILIs), five emergency department visits and three specialist visits – evenly distributed between the cohorts (data not shown). No hospitalisations were reported. Antibiotics were used for 52 ILIs and 73 were treated with analgesic/antipyretics – evenly distributed between the cohorts (data not shown). The median duration of ILIs was 8 days, but 16 (13%) lasted more than 28 days.

Forty-four ILI episodes (35%) met our definition of ‘severe ILI’: 15 had otitis media, 31 had symptoms other than post-ILI dry cough persisting >14 days, and 9 had fever persisting ≥5 days (some overlap).

### Virus identification

Swab samples were available for 117 (94%) of the 124 ILI episodes, both nose and throat (69 ILIs) or nose only (48 ILIs).

**Figure 1:**
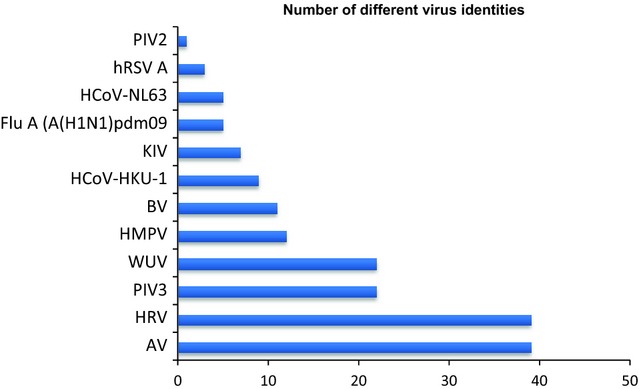
Number of different virus identities.

**Figure 2:**
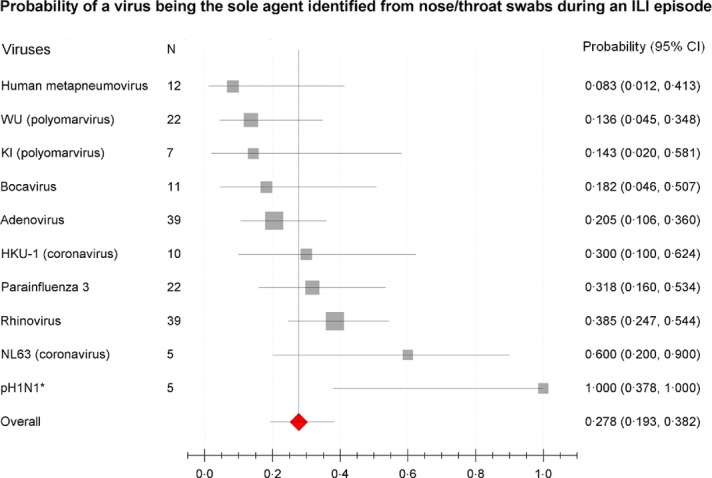
Probability of a virus being the sole agent identified from nose/throat swaps during an ILI episode. N, number of times identified; *Influenza virus A(H1N1)pdm09: pandemic influenza A/California/7/2009 (H1N1).

The quality of samples was high in terms of extraction and cell collection; only one sample failed EHV testing and ERV3 was detected in all but one specimen, and this specimen was negative of all other viruses. A total of 175 viruses were identified from 103 ILIs (see Figure 1). Multiple viruses were detected in 52 (44%) of the swabbed ILIs – 38 ILIs yielded two viruses each, nine yielded three viruses, four yielded four, and one yielded five. The probability of a virus being the sole agent identified from nose/throat swabs during an ILI episode is shown in Figure 2. Influenza A(H1N1)pdm09, which was the sole virus causing 5 ILIs, was the only virus consistently identified as the sole agent from all ILIs with which it was associated. Although coronavirus NL63 (3 of 5 ILIs in which they were identified) and rhinovirus (15 of 39 ILIs in which they were identified) were frequently identified as sole agents of ILIs, that tendency was not statistically different to the probabilities of the other non-influenza viruses being solely identified.

No particular virus or virus combination or multiplicity of virus infection was associated with any particular symptom or combination of symptoms or with greater frequency of antibiotic or analgesic/antipyretic use, GP visits or other healthcare service usage. One or more of the polyomaviruses WUV and KIV were more commonly identified in children aged <2 years (*P* = 0·05), and adenoviruses were more common in females (*P* = 0·03) – data not shown. Rhinovirus alone or in combination with other viruses was associated with longer duration of ILI than other viruses (*P* = 0·02). None of these values were corrected for multiple testing.

Of the five children who had influenza A(H1N1)pdm09 infection, one was fully vaccinated, one was partially vaccinated (1 dose of Panvax, CSL, in October 2009), and three were not vaccinated against influenza. The management of the A(H1N1)pdm09 infections required GP visits for four of the children; three received antipyretic/analgesic medications, and two received antibiotics.

### Yield of viruses by swabbing site

Nose swabs were collected from 117 swabbed ILIs, while throat swabs were collected from 69. The use of neither nose nor throat swabs was not significantly differently distributed across the three cohorts. Furthermore, there was no statistical difference in the number of throat swabs collected from the three study groups (*P* = 0·20). Swabs were not combined prior to testing. One or more viruses were detected in 88% of swabbed ILIs. Nose swabs more often yielded viruses – 102/117 (87%) – than did throat swabs – 45/69 (65%), *P* < 0·001.

Nose swabs yielded more viruses per swab than did throat swabs (161 virus identities from 117 nose swabs = 1·38 viruses/swab compared to 59 virus identities from 69 throat swabs = 0·86 viruses/swab, *P* < 0·001). Limiting the comparison of virus yields from nose versus throat swabs to ILIs from which both nose and throat swabs were taken (*n* = 69) gave the same rates of virus identification (1·37 viruses per swab for nose swabs, 0·86 viruses per swab for throat swabs, *P* = 0·001) with 61 (88%) of 69 nose swabs yielding a virus and 45 (65%) of 69 throat swabs yielding a virus, *P* = 0·002.

### ILI transmission within households

In the week before onset of their ILIs, only nine subjects were exposed to one or more household members with ILIs (three other children and eight adults). In the week after onset of the subject's ILI, eight other children (154 exposed, 5% attack rate) and 38 adults (244 exposed, 16% attack rate) in the ill subjects' household reported ILIs. Adult household members more often developed an ILI in the week after ILI onset in subject children than did child members of the households, *P* = 0·001 (asymptomatic carriage and transmission were not taken into account as it could not be identified). No virus was more likely than any other to be transmitted from the ill subject to members of the household.

## Discussion

In the 2010 Southern Hemisphere influenza season in Sydney, Australia, we found that young children suffered relatively often from ILIs, but less than in previous studies.^20^ The ILIs were caused by many different viruses, most commonly rhinoviruses and adenoviruses. Adenovirus was more commonly found in females, an association which has not been reported previously^21^,^22^ and may be due to chance as no correction for multiple testing was performed. In contrast to others, we detected few RSV infections^14^,^23^,^24^,^20^ probably because RSV infections peaked in Sydney during June and July 2010 and had declined significantly in frequency by the time we commenced observations for ILIs in the study participants (from 30 July 2010).

We found only 5 ILIs caused by influenza viruses – all A(H1N1)pdm09. Their illnesses were little different to the ILIs experienced by the children from whom other viruses were identified (data not shown). However, the small number of influenza infections is consistent with the low degree of influenza activity during 2010,^25^ limiting the power of this study to detect differences in influenza infection rates.

We did, however, unexpectedly find that non-influenza ILI occurred about 1·6 times more commonly in children vaccinated with one or two doses of the influenza vaccine than in unvaccinated children. These results support the findings of a recent RCT reported by Cowling *et al*.^26^ Cowling's study in Hong Kong concluded that non-influenza ARI may be detected at a higher rate in children for a short period after they received influenza vaccine. The non-influenza virus incident rate ratio (IRR) was higher in the Hong Kong study (4·4 versus 1·6), but there are some key differences to our study, including age of subjects, follow-up period, proportion of illnesses swabbed and proportion of swabs yielding viruses. As with all observational studies, bias must be considered. Vaccinated and unvaccinated cohorts in Sydney were demographically similar (Table 1); however, lack of blinding by vaccination status makes it difficult to rule out selection or measurement bias. We could find no evidence of different parental responses to ILIs in vaccinated and unvaccinated children: parents of vaccinated children were no more likely to seek medical care during an ILI. However, we did find that health-seeking behaviours, recorded on enrolment (before the ILI observation period), such as hospitalisation (any cause), hearing tests and grommet insertion were significantly more common in the vaccinated groups, suggesting that families that vaccinate children have a prior preference for greater healthcare service usage. This may be a partial explanation for the observed difference in ILI frequency between the groups; however, prior access to any of these healthcare services did not predict the frequency of reported ILI.

Cowling *et al*. proposed possible explanations ranging from an unknown biological mechanism by which vaccine-induced immunity to influenza was accompanied by decreased immunity to other respiratory virus to a temporary non-specific immunity (interferon- and/or cell-mediated related) to other respiratory viruses after wild influenza infection. A formal biological explanation is lacking. A recent US observational (case–control) study has not found an association between influenza vaccination and detection of non-influenza respiratory viruses^27^. Re-analysis of observational studies or preferably new RCTs with high parent-collected specimen availability is required to further examine this phenomenon. It should be a priority to determine whether a causal association exists, whether it is consistent across vaccines and populations and whether any observed increase in the rate of non-influenza respiratory virus identification outweighs the benefit of seasonal TIVs in children.

In nearly half (44%) of the nose/throat swabs, multiple viruses were detected. Other studies in different settings, using a variety of definitions for ILI, have reported diverse virus aetiologies for ILIs, but, in general, with lower frequencies of virus co-infection than we report.^14^,^23^,^24^,^20^,^28^ Our finding may be explained by the large number of viruses for which we tested and also by our high rate of swabbed ILIs being positive (88%).

While we found no increased severity or number of symptoms with multiple virus compared with single virus infection, this study lacked power to tease apart the relative significance of each virus, virus combinations and multiplicity of infection. A simultaneously sampled asymptomatic control group would have been useful to explore further the meaning of multiple virus identification. Interestingly, influenza A(H1N1)pdm09 was the only virus constantly identified as the sole virus from ILIs. However, there were too few cases (five only) to permit a firm conclusion about this.

We found that nose swabs were more effective than throat swabs for detecting respiratory viruses in young children with ILIs. Also, parents more frequently collected nose swabs than throat swabs from their children, suggesting that they may be more acceptable.

In our study, we detected the polyomaviruses WUV and KIV at higher frequencies (mainly as co-infections) than other investigators.^29^–^38^ Children aged <2 years were more often infected than older children, but this barely reached a statistical significance. To date, the pathogenicity and clinical significance of WUV and KIV (discovered in 2007)^3^,^4^ remain unclear and studies, conducted in a variety of settings and with a variety of respiratory disease definitions, have yielded inconsistent results.

Key strengths of our study include the high rate of specimen collection (94% of ILIs were swabbed) and the high rate of virus detection in swabbed ILIs (88% of all cases swabbed yielded at least one virus). We believe that parent-collected specimens combined with mail return of the specimens to the laboratory can be considered a reliable means of virus detection for studies such as ours, partly because children are sampled earlier when viral loads may be higher.

### Limitations

The participant characteristics are somewhat different to the general Australian population. Participating households had a higher income (83% of the studies households compared to 30% of Australians had income of $ 2000/week or more),^39^ the mothers were slightly older (33·1 years compared to 30·1 years in the general population)^40^ and were more likely to live with a partner (married or de facto 97% compared to 88%)^41^ and, because we recruited in CCCs, 89% of the study population were in formal child care compared to 35% of Australian children aged 0–4 years.^42^ At the start of the ILI follow-up period, we established that all parents/guardians were receiving reminder messages, and during the course of the ILI follow-up period, we spoke to parents/guardians of 52% of the enrolled children (evenly distributed across the cohorts – data not shown). However, we did not contact all parents/guardians after the ILI follow-up period in order to determine whether they had not reported ILIs. We did record at enrolment data on prior healthcare service usage (e.g. hospitalisation) and this was higher in vaccinated children; all this might have biased the research to show a higher frequency of ILI in influenza vaccinees. The open-label cohort design is also open to unmeasured confounding.

## Conclusion

Influenza-like illness is common in children, and the burden on their families may be considerable. Many different respiratory viruses are responsible for ILIs in children. In this study, conducted after the RSV season, adeno- and rhinoviruses were the most commonly detected viruses. Symptom profiles were similar among the different viruses, and the rate of virus co-infection was high. Recipients of influenza vaccines had about 1·6 times more ILI episodes than did unvaccinated children, and although this may be at least partly explained a healthcare service-seeking bias, further investigations are warranted into whether influenza vaccine increases the risk of non-influenza ILI, as healthcare-seeking behaviour did not predict ILI in a regression model.

Nose swabs collected by parents had a high yield of respiratory viruses when using multiplex PCR methods and had significantly more viruses compared to throat swabs. In addition, parents appeared to feel more comfortable in performing nose than throat swabs. This is of relevance to future studies requiring parent-collected samples for PCR analysis.

## Addendum – List of Authors

Dr. Alexa Dierig contributed substantially to the design of the study, helped with analysis and interpretation of data and wrote and revised the intellectual content. She was also the study co-ordinator. Dr. Leon Heron contributed substantially to the concept and design of the study, helped with analysis and interpretation of data and wrote and revised the intellectual content. A/Prof Stephen Lambert contributed substantially to the concept and design of the study, helped with analysis and interpretation of data and wrote and revised the intellectual content. Dr. Jiehui Kevin Yin analysed the data, helped with their interpretation and revised the intellectual content. A/Prof Julie Leask contributed substantially to concept and design of the study and revised the intellectual content. Maria Yui Kwan Chow helped with analysis of data and revised the intellectual content. Prof Theo Sloots contributed substantially to the concept and design of the study and helped with the analysis of data. Prof. Michael Nissen contributed substantially to the concept and design of the study and helped with the analysis of the data. Dr Iman Ridda helped with the interpretation of data and wrote and revised the clinical content. Prof Robert Booy contributed substantially to concept and design of the study, helped with analysis and interpretation of data and also with writing and revising of the intellectual content. He was the supervisor of the whole project. All authors approved the final version.
